# Study on Antibacterial Powder Coatings Based on Halloysite/Biopolymer Compounds

**DOI:** 10.3390/ma18235402

**Published:** 2025-11-30

**Authors:** Katarzyna Krawczyk, Barbara Pilch-Pitera, Michał Kędzierski, Małgorzata Zubielewicz, Izabela Kunce, Ewa Langer, Sebastian Jurczyk, Grażyna Kamińska-Bach, Ewa Ciszkowicz, Marta Przybysz-Romatowska, Damian Wojda, Leszek Komorowski, Michael Hilt

**Affiliations:** 1 Fraunhofer Institute for Manufacturing Engineering and Automation, 70569 Stuttgart, Germany; katarzyna.krawczyk@ipa.fraunhofer.de (K.K.); michael.hilt@ipa.fraunhofer.de (M.H.); 2 Faculty of Chemistry, Rzeszow University of Technology, Powstańców Warszawy 6, 35-029 Rzeszów, Poland; ewa.ciszkowicz@prz.edu.pl; 3 Łukasiewicz Research Network—Industrial Chemistry Institute, Rydygiera 8, 02-724 Warsaw, Poland; michal.kedzierski@ichp.lukasiewicz.gov.pl (M.K.); marta.przybysz-romatowska@ichp.lukasiewicz.gov.pl (M.P.-R.); 4 Łukasiewicz Research Network, Institute for Engineering of Polymer Materials and Dyes, 87-100 Toruń, Poland; malgorzata.zubielewicz@impib.lukasiewicz.gov.pl (M.Z.); ewa.langer@impib.lukasiewicz.gov.pl (E.L.); sebastian.jurczyk@impib.lukasiewicz.gov.pl (S.J.); grazyna.kaminska-bach@impib.lukasiewicz.gov.pl (G.K.-B.); 5 Road and Bridge Research Institute, 03-302 Warsaw, Poland; damian.wojda@ibdim.edu.pl (D.W.); leszek.komorowski@ibdim.edu.pl (L.K.)

**Keywords:** halloysite, polylysine, quaternized chitosan, antimicrobial properties, polyester coatings, powder coatings, biopolymers, antibacterial activity, *Escherichia coli*, *Staphylococcus aureus*

## Abstract

This study presents an eco-friendly approach to antibacterial polyester powder coatings by incorporating hybrid additives composed of biopolymers immobilized on halloysite nanotubes. Polylysine (PLY) and quaternized chitosan (CH-Q) were used as natural antimicrobial agents, while halloysite (HAL) acted as a carrier to improve dispersion and reduce leaching. HAL/PLY and HAL/CH-Q hybrids were incorporated into polyester coatings and evaluated for morphology, mechanical properties, water resistance, and antibacterial performance (ISO 22196). The HAL/PLY coating demonstrated a strong bactericidal effect, reducing Escherichia coli and Staphylococcus aureus by 99.9989% and 99.9993%, respectively. HAL/CH-Q showed moderate activity against *E. coli* (50.2323%) but high activity against *S. aureus* (98.6500%). Immobilization of biopolymers on the halloysite surface improved dispersion and barrier properties while enabling a silver-free antibacterial effect. The results demonstrate a sustainable strategy for multifunctional powder coatings based on naturally derived antimicrobial components.

## 1. Introduction

The demand for polymeric materials and coatings capable of inhibit microbial growth has increased significantly in recent years, driven by their widespread use in healthcare, public spaces, consumer products, and the food industry [1,2,3,4].

Antimicrobial technologies are expected to not only reduce bacterial and viral contamination but also comply with strict environmental and safety standards. Conventional biocides often raise concerns about long-term toxicity, environmental persistence, and the risk of promoting antimicrobial resistance. Selective action, limited bioaccumulation, and a reduced ecological footprint remain key criteria for next-generation antimicrobial materials [5].

Antimicrobial coatings generally rely on three principal mechanisms: (i) biocide-releasing systems where active agents diffuse from the coating; (ii) contact-killing surfaces, usually utilizing cationic or reactive functionalities; and (iii) anti-adhesion coatings that hinder microbial attachment and biofilm development [6,7]. Each strategy has its benefits but also faces challenges like uncontrolled leaching, limited short-term effectiveness, or poor compatibility with polymer matrices. Recent studies comparing antimicrobial polymers stress the importance of sustainable, metal-free options and point to the potential of cationic biopolymers and hybrid fillers as alternatives to silver-based systems [8].

A comprehensive analysis by Mölling et al. [9] revealed that more than half of 23 commercial antimicrobial coatings relied on nanosilver and achieved strong antibacterial performance (up to ~6-log reduction) according to ISO 22196 [10]. Similar activity was observed in systems with zinc- or TiO_2_-based modifiers, organoclays, or quaternary ammonium compounds. However, despite their high effectiveness, these methods face growing regulatory limits and environmental issues. In the case of silver, concerns include bioaccumulation, immunotoxicity, and possible disruption of soil microbial ecosystems. [11,12]. Repeated exposure has even been reported to induce bacterial resistance to silver nanoparticles [13]. Triclosan, another widely used antimicrobial agent, accumulates in the environment, interferes with endocrine function, and can photodegrade into toxic dioxins under sunlight exposure [14]. Similarly, TiO_2_-based photocatalytic systems exhibit strong antimicrobial activity only under specific environmental conditions, such as sufficient UV intensity and humidity, and typically show reduced performance under visible-light irradiation [15].

These constraints highlight the increasing importance of bio-based, low-leaching, and environmentally friendly antimicrobial strategies.

Biopolymers like polylysine (PLY) and quaternized chitosan (CH-Q) are promising candidates because of their natural origin, cationic nature, and well-known antimicrobial mechanisms that include membrane disruption and electrostatic interactions [15]. Our earlier research showed that immobilizing chitosan on layered silicates significantly reduces its leaching from polymer coatings [16], indicating that solid-support strategies can improve both the stability and effectiveness of biopolymer-based systems.

In this context, halloysite nanotubes (HAL) were selected as natural aluminosilicate carriers for biopolymer immobilization. Halloysite (Al_2_Si_2_O_5_(OH)_4_·nH_2_O) consists of alternating alumina and silica layers rolled into hollow tubular structures. It exhibits good biocompatibility, favorable dispersion behavior in aqueous and polymeric systems, and may possess intrinsic antibacterial activity due to Fe(II) species frequently present in natural deposits [17,18,19]. These characteristics make halloysite a suitable platform for creating hybrid antimicrobial additives that combine the advantages of biopolymers with those of inorganic nanostructures.

The goal of this work is to develop environmentally friendly polyester powder coatings with halloysite–biopolymer hybrids based on PLY and CH-Q. It also aims to evaluate how immobilization on halloysite affects dispersion in the polyester matrix, as well as barrier, mechanical, and antibacterial properties. This study aims to demonstrate a sustainable, silver-free antimicrobial approach for multifunctional powder coatings.

## 2. Materials and Methods

### 2.1. Antimicrobial Agents (AA): Materials and Preparation Methodology

ε-Polylysine (PLY, >95%) was supplied by Mark Nature (Fullerton, CA, USA). Chitosan (90/10/A1, 90% deacetylation degree, viscosity 10 cps) was supplied by BioLog Heppe GmbH (Queis, Germany). Halloysite was obtained from the Dunino mine in Poland.

Before each experiment, the halloysite powder was sonicated in water. For this purpose, 300 g of halloysite was dispersed in approximately 1.5 l of distilled water, stirred for 10–15 min, and placed in a 35 kHz ultrasonic bath while mechanically stirred for 3 h. After sonication, the material was decanted and dried in an oven at 90 °C for 12 h.

Quaternized chitosan (CH-Q) was synthesized through the following process: in a 2 L reactor equipped with a reflux condenser and a stirrer, 700 mL of water, 70 g of chitosan, and 140 g of glycidyltrimethylammonium chloride (GTMAC) were added. While stirring, the mixture was heated to 80 °C and maintained at this temperature for 10 h. A darkened mass with the consistency of thick jelly formed. After cooling, an aqueous hydrochloric acid solution was added to adjust the pH to 5–6. Then, 1 L of acetone was added, the precipitate was separated, and it was washed with acetone while stirring for 1 h at room temperature. The product was then filtered and dried in a vacuum oven at 40 °C.

To immobilize polylysine (PLY) on halloysite, 15 g of halloysite was suspended in 50 mL of water and ultrasonically stirred for 1 h. Simultaneously, 7.5 g of PLY was dissolved in 150 mL of water. Both solutions were combined, stirred for 2 h at room temperature, and left to stand overnight. The precipitate was filtered and dried under vacuum. The same method was used to immobilize quaternized chitosan (CH-Q) on halloysite.

To enhance the activity of HAL/CH-Q compounds, they were additionally modified by ultrasound in an aqueous suspension (10 g in 40 mL of water, 80% amplitude, cycle 1.0, for 18 min) and dried at 80 °C under vacuum.

### 2.2. Preparation of Powder Coatings

The following raw materials were used to prepare the powder paint:

- Saturated polyester resin: GP 95518, acid value: 35 mg KOH/g (Sarzyna Chemical, Sarzyna, Poland);
- β-hydroxyalkyl-amide curing agent: Primid XL-552, hydroxyl number: 620–700 mg KOH/g, melting range 120–125 °C (EMS-CHEMIE AG, Domat/Ems, Switzerland);
- Degassing agent: benzoin (Aldrich, Buchs, Switzerland);
- Flow control agent: Byk 368P (Byk-Chemie, Wesel, Germany);
- Filler: barium sulfate, Albasoft 100 (Deutsche Baryt-Industrie, Bad Lauterberg, Germany);
- Brown iron pigment: Bayferrox 654 T (Lanxess, Köln, Germany);
- Antimicrobial agents: ε-polylysine (PLY), halloysite (HAL), PLY immobilized on halloysite (HAL/PLY), quaternized chitosan (CH-Q) or quaternized chitosan (CH-Q) immobilized on halloysite (HAL/CH-Q).

The composition of the powder coatings is presented in Table 1.

All raw materials were weighed and mixed, then ground and extruded using a co-rotating twin screw mini extruder EHP 2 × 12 Sline (Zamak, Crakow, Poland). The extrusion temperatures were zone I-60 °C, zone II-90 °C, zone III-100 °C, and adapter-115 °C. The screw’s rotational speed was 150 rpm. After extrusion, the cooled extrudates were pulverized, and sieved through a 100 μm sieve.

The powder coatings were applied to the matt steel substrate (Q-Panels R-36) and glass plates using a CORONA spray gun (PEM X-1) controlled by an EPG Sprint X unit (Wagner, Alstatten, Switzerland). The Q-panels, made from standard low-carbon cold-rolled steel, were taken directly from sterile packaging and used according to the manufacturer’s recommendations. Glass plates were washed with acetone immediately before coating. All coated samples were cured at 160 °C for 15 min.

### 2.3. Measurements

#### 2.3.1. Characterization of Antimicrobial Agent

The UNICUBE/Rapid OXY Cube elemental analyzer (ELEMENTAR Analysensysteme, Langenselbold, Germany) was used determine carbon, hydrogen, and nitrogen content. The analysis temperature were combustion tube 115 °C, reduction tube 85 °C, adsorption column standby 40 °C, and cooling 90 °C. Time parameters were: purge function 45 min, O_2_ injection delay—20 min, and integrator reset delay for peak N—10 min, for peak C—1 min, for peak H—1 min, integrator reset delay for peak S—2 min. The analysis was performed on three replicates of the same sample. The result was the arithmetic mean of the three analyses, and the standard deviation was calculated.

Thermogravimetric analysis (TGA) was performed using TGA/SDTA 851e instrument (Mettler-Toledo, Greifensee, Switzerland) in the temperature range from 25 to 600 °C, with a heating rate of 10 °C min^−1^, under nitrogen atmosphere (50 mL·min^−1^). Approximately 5 mg of sample was placed in 150 μL open alumina pans.

The morphology and microstructure of the antimicrobial additives were characterized using a scanning electron microscope (JEOL JSM-6010LV, JEOL, Tokyo, Japan) in backscattered electron (BSE) imaging mode at low vacuum.

Powder X-ray diffraction (PXRD, Bruker AXS GmbH, Karlsruhe, Germany) spectra were recorded on a MiniFlex600 powder diffractometer, Rigaku, with the following parameters: radiation: CuK_α1_, λ = 1.54056 Å, scanning range 2q: 2–80°.

FTIR analysis was performed using a spectrometer Nicolet iS10 Thermo Scientific (ThermoFisher Scientific, Waltham, MA, USA). The ATR/diamond crystal technique was used to obtain spectra of the coating surface.

#### 2.3.2. Characterization of Powder Coatings

A Mar Surf PSI profilometer (Mahr, Göttingen, Germany) was used to measure the roughness of cured powder coatings in accordance with ISO 12085 [20].

A micro-TRI-gloss µ tester from BYK-Gardner GmbH (Geretsried, Germany), was used to determine the gloss and thickness of the cured powder coatings in accordance with ISO 2813 [21] and ISO 2808 [22], respectively. The gloss was measured at 60° angle.

Adhesion to the steel surface was evaluated by a cross-cut test by using BYK-Gardner, (Geretsried, Germany) according to ISO 2409 [23].

König Pendulum tester from BYK-Gardner GmbH (Geretsried, Germany) was used to measure the relative hardness of the cured powder coatings according to ISO 1522 [24].

The cupping was determined according to the ISO 1520 standard [25]. The tests were performed using a manual SP4300 tester by TQC (Capelle aan den Ijssel, Netherlands). The spherical drawing punch was used to indent a clamped sheet until the coating cracked. The point of crack initiation was then recorded. To assess the repeatability of the results, three measurements were conducted on the same cured coating.

Differential scanning calorimetry (DSC) was performed using a DSC 1 Instrument (Mettler-Toledo, Greifensee, Switzerland). Samples of 7–9 mg, in the form of disks of 5 mm diameter, cut as 5 mm disks from the free coatings, were heated from 0 °C to 200 °C at 20 °C·min^−1^ and cooled at the same rate under nitrogen (60 mL·min^−1^). Samples were sealed in a 40 µL aluminum crucibles. Glass transition temperatures were determined during the second heating run. Data was evaluated using STAR^e^ Mettler-Toledo v.15 software.

Dynamic mechanical analysis (DMA) was carried out using a DMA 861e (Mettler-Toledo, Greifensee, Switzerland) to measure the storage modulus, loss modulus, and tan δ. The samples, in the form of disk-shaped specimens taken from free coatings, were heated from −65 °C to 150 °C at 3° C·min^−1^. The shear mode was used to the test the coatings with a displacement amplitude of 1 µm and a frequency of 1 Hz. Data were processed using STARe Mettler-Toledo v.15 software.

Color parameters were determined using the CM-2600d spectrophotometer (Konica Minolta Sensing Inc., Osaka, Japan) in accordance with ISO 7724 [26].

Measurements were performed using measurement geometry d:8°, standard observer 10°, CIE D65 standard illuminant in SCE (Specular Component Excluded) mode, with a spectral interval of 10 nm. Color evaluation was based on the CIELAB color space parameters (L*, a*, b*), where

- L*—lightness (0 = black, 100 = white),
- a*—green-red axis (− = green, + = red),
- b*—blue-yellow axis (− = blue, + = yellow).

The color difference (ΔE*_ab_) was calculated according to Equation (1):

(1) $$\Delta {E*}_{ab}=\sqrt{{\Delta L*}^{2}+\Delta {a*}^{2}+{\Delta b*}^{2}}.$$

The results of the average values of the specified coating properties (mechanical or visual) are given together with the standard deviation. To determine whether the differences between the average values of specific coating properties are statistically significant, where applicable, a t-test was performed to compare the data values with the reference value. Three *p*-value ranges were used to determine the differences between the values: *p* < 0.05 (statistically significant), *p* < 0.01 (highly significant) and *p* < 0.001 (very highly significant).

Antibacterial activity of the coating surfaces was evaluated according to ISO 22196 [10] against *E. coli* and *S. aureus* bacteria. Bacterial strains were cultured, undergoing at least two passages at 37 °C in a New Brunswick Innova 40 Shaker (Eppendorf AG, Hamburg, Germany) to ensure consistent growth. Cultures in logarithmic phase were standardized against the 0.5 McFarland (corresponding to 1–5 × 10^8^ colony forming units/mL, CFU/mL) by measuring OD_600_ with a Varioskan™ LUX multimode microplate reader (Thermo Scientific™, Waltham, MA, USA). The final bacterial inoculum was prepared by mixing 1 mL of the prepared bacterial culture (1–5 × 10^8^ CFU/mL) with 100 mL of agar suspension at 45 ± 2 °C, reaching a final density of approx. 10^6^ CFU/mL. The tested coatings were placed in Petri dishes and moistened with saline solution using sterile cotton plugs. Subsequently, 0.5 mL of the inoculated agar suspension (bacterial inoculum) was applied to each coating, forming a “pseudo-biofilm” layer not exceeding 1 mm. After solidification, the coatings were incubated for 24 h at 37 °C in a high-humidity incubator. Following incubation, control and test samples were transferred to sterile beakers using flame-sterilized tweezers. Each beaker (“A”) received 4.5 mL of neutralizing broth, yielding a 1:10 dilution. The beakers were sealed with parafilm, sonicated, and vortexed for 1 min. A series of 10-fold dilutions was then prepared: 4.5 mL of neutralizing broth was added to 15 mL Falcon tubes (“B”, “C”, “D”), and 0.5 mL of the bacterial suspension was successively transferred from “A” → “B” → “C” → “D”, resulting in a final dilution of 1:10,000.

For inoculation, each agar plate was divided into three sections, into which 25 μL of the appropriate solution was applied and spread evenly using sterile spreaders. The samples were incubated at 37 °C for 24 h. After incubation, colony-forming units (CFU) were counted. Antibacterial performance was expressed as the reduction in bacterial count compared with the reference coating (without antimicrobial additive). For each strain, results represent the mean of three independent samples as technical replicates.

After solidification, the coatings were incubated for 24 h at 37 °C in a high-humidity incubator.

A Scanning Kelvin Probe (SKP) system (Anfatec Instruments AG, Oelsnitz, Germany) was employed to measure the Volta potential distribution on the surface of the cured powder coatings. Measurements were conducted under controlled laboratory conditions (T = 22 ± 2 °C, RH = 45 ± 5%) in a dry atmosphere. The tip-to-sample distance was set to approximately 20 µm. The SKP technique was used to evaluate surface potential uniformity and to identify local heterogeneities related to filler distribution or interfacial phenomena at the coating–substrate interface. The obtained potential maps were processed using Anfatec MultiKelvin Software Revision 5.0 to generate 2D potential images and corresponding line profiles.

Water Uptake Reversibility (WUR)—Capacitance Monitoring: the water uptake behavior of the cured powder coatings was evaluated by cyclic monitoring of coating capacitance using Electrochemical Impedance Spectroscopy (EIS) with a Gamry Reference 600+ potentiostat (Gamry Instruments, Warminster, PA, USA). Measurements were carried out under sinusoidal temperature cycling between 20 °C and 50 °C, with one complete cycle per hour, for a total duration of 4 h. The coating capacitance C was determined from the impedance spectra at a fixed frequency of 100 kHz according to Equation (2):

C = ε_0_·ε_r_·A/d, (2)

where

C: coating capacitance,
ε_0_: vacuum permittivity (8.854*10^−12^ F m^−1^),
A: detected area,
d: coating thickness,
ε_r:_ dielectric constant, ε_r_ (H_2_O) ≈ 80, ε_r_ (polymer) < 8.

Since the dielectric constant of water (ε_r_ (H_2_O) ≈ 80) is much higher than that of the polymer matrix (ε_r_ (polymer) < 8), an increase in coating capacitance indicates water penetration into the coating. The amplitude of the cyclic capacitance change (ΔC) was used as a comparative measure of water uptake reversibility for different coatings.

#### 2.3.3. Washing Resistance of Antibacterial Additives

The leachability of antibacterial additives from powder coatings was assessed by measuring the electrical conductivity of the aqueous solution at different time intervals. Coating samples of similar dimensions and mass were prepared and placed in laboratory containers. A specific volume of deionized water (60 mL) was added to each sample. The containers were tightly sealed and kept at room temperature.

Electrical conductivity was measured using a Microcomputer Conductivity Meter (Elmetron CC-551, Zabrze, Poland). Conductivity was recorded at designated time intervals—after 24 h, 96 h, 7 days, and so on. The samples remained immersed in water for 2 months. The conductivity electrode was rinsed with deionized water before and after each measurement. The study was conducted at room temperature (approximately 22–25 °C), and temperature fluctuations were monitored, as the electrical conductivity of water is temperature-dependent.

## 3. Results and Discussion

### 3.1. Antimicrobial Agents’ Characterization

Halloysite (HAL) and biopolymers such as polylysine (PLY) and quaternized chitosan (CH-Q) were employed to modify powder coatings for antibacterial activity. Polylysine (PLY) and quaternized chitosan (CH-Q) were used in their original form and as an immobilization product on halloysite HAL/PLY and HAL/CH-Q.

Halloysite consists of platy and tubular structures made of silicon dioxide tetrahedra and aluminum oxide octahedra. The Polish halloysite from the Dunino mine used in our work also contains also significant amounts of iron as hematite Fe_2_O_3_ and magnetite Fe_3_O_4_, which is indicated by the brownish color of the mineral [27].

**Figure 1:**
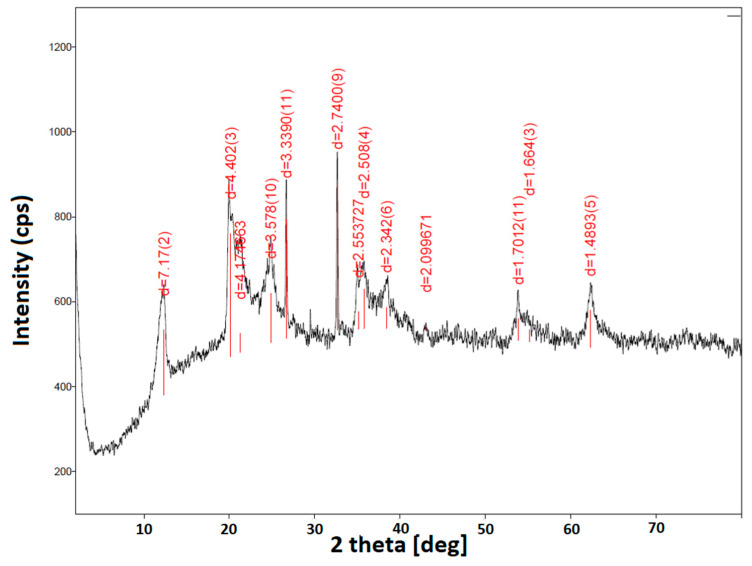
XRD pattern of halloysite HAL.

The XRD pattern of the halloysite used in this study is shown in Figure 1. It displayed prominent peaks at 2θ of 12.34°, 20.15°, and 24.87°, corresponding to d-spacings of 7.2, 4.4, and 3.6 Å, respectively, representing the (001), (100), and (002) reflection planes indexed to ICDD (International Centre for Diffraction Data) 00-029-1487 [28].

The position of the (001) diffraction peak indicates the dehydrated form of halloysite, known as halloysite-7 Å, with the chemical formula Al_2_Si_2_O_5_(OH)_4_, which is usually found in dry climates [29]. The reflection at 2θ = 43.14°, corresponding to d = 2.099 Å, based on nanobeam electron diffraction (NBD/SAED) can be assigned to the (200) plane of magnetite [[30], ICDD 00-019-0629].

**Figure 2:**
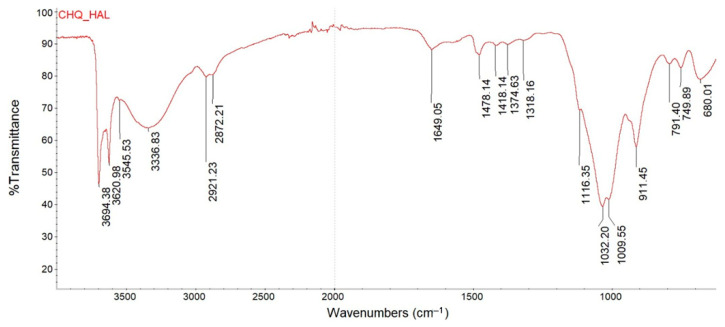
FTIR spectrum of quaternized chitosan intercalated on halloysite.

Figure 2 shows the FTIR spectrum of quaternized chitosan intercalated on halloysite. You can see characteristic bands confirming the presence of chitosan and halloysite [31]. The Al_2_O-H stretching bands at 3694 and 3620 cm^−1^, hydrogen bonds assigned to the OH groups near 3300 cm^−1^, an intensive Si-O stretching band at 1009 cm^−1^, signals at 911 cm^−1^ and in the range of 787–749 cm^−1^ are related to OH groups originating from halloysite. Hydrogen bond assigned to OH groups (3330 cm^−1^), the signals at 2921 cm^−1^ and 2872 cm^−1^ are attributed to C-H stretching vibration of aliphatic groups; the signal at 1649 cm^−1^ is related to -NH_2_; the small absorption band near 1116 cm^−1^ is attribute to the ester groups C-O-C. The signal at 1478 cm^−1^ is related to C-H asymmetric bending vibration of N^+^(CH_3_)_3_ group and it confirms successful chitosan quaternization [32]. The presence of positively charged ammonium substituents is crucial for the antimicrobial activity of quaternized chitosan.

Combustion elemental analysis of AA agents (Table 2) shows that the nitrogen content and the nitrogen-to-carbon weight ratio are considerably higher in PLY than in CH-Q, which can be attributed to the higher oxygen content in the CH-Q chain. The carbon and nitrogen content in halloysite is very low because it is composed mainly of aluminosilicates. The HAL/PLY and HAL/CH-Q immobilization products exhibit nitrogen contents of 5.22 ± 0.03% and 3.34 ± 0.01%, respectively, and these differences significantly influence their antibacterial properties.

**Figure 3:**
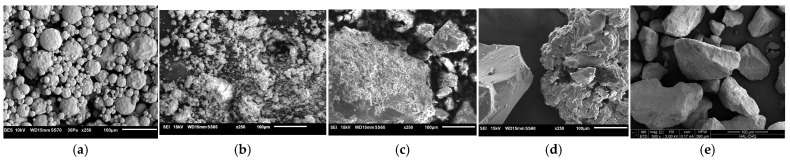
SEM morphology of antimicrobial additives: (**a**) polylysine (PLY), (**b**) halloysite (HAL), (**c**) polylysine immobilized on halloysite (HAL/PLY), (**d**) quaternized chitosan (CH-Q) and (**e**) quaternized chitosan immobilized on halloysite (HAL/CH-Q).

The morphology of antimicrobial additives used in the fabricated powder coatings is shown in Figure 3. Polylysine appears as spherical particles with a slightly developed surface and particle size ranges from 5 to 100 μm. The quaternization process of chitosan causes changes on the surface of most particles. Figure 3d shows the original chitosan particles with a compact structure and particles after modification through quaternization. As a result, the structure of the particles becomes more developed, and many agglomerates, composed of particles ranging from 5 to 50 μm, are formed. Quaternized chitosan immobilized on halloysite does not form agglomerates; instead, chitosan molecules cover the surface of halloysite.

**Figure 4:**
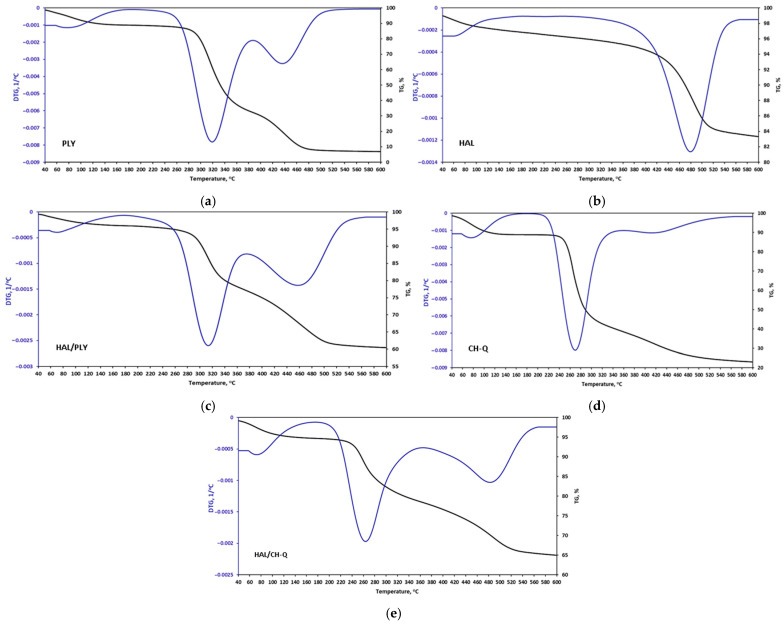
TGA curves of antimicrobial additives: (**a**) polylysine (PLY), (**b**) halloysite (HAL), (**c**) polylysine immobilized on halloysite (HAL/PLY), (**d**) quaternized chitosan (CH-Q) and (**e**) quaternized chitosan immobilized on halloysite (HAL/CH-Q).

To evaluate whether the developed antimicrobial components are thermally stable under the conditions of powder coating processing and curing, thermogravimetric analysis (TGA) was performed. The results were presented in Figure 4 and in Table 3.

The initial stage of thermal decomposition of the raw materials and halloysite involves the desorption of water that is adsorbed and bound on the surface and within the interlayer space of the aluminosilicate. The water content was highest for CH-Q (10%) and lowest for HAL (2.5%). In the case of halloysite, the next mass loss stage occurs between 380 and 540 °C, corresponding to structural dehydroxylation processes [29].

The TGA of polylysine shows two distinct decomposition steps: one occurring between 180 and 380 °C and the other above 380 °C, with mass losses of 65% and 26%, respectively. Quaternized chitosan also undergoes a two-step decomposition. The first phase occurs at 180 °C, and the second above 400 °C, corresponding to the dehydration of the monomer units, depolymerization, and finally the decomposition of the chitosan molecule [33].

For PLY, a lower char residue is observed (9%), while HAL exhibits 15% weight loss, which may be related to its high thermally stable aluminosilicate structure. As a result of PLY or CH-Q immobilization on HAL, an increase in the temperature of the DTG peaks associated with polymer decomposition is observed, indicating improved thermal stability.

Based on the TGA thermograms, all tested antimicrobial additives are thermally stable up to about 180 °C. Within this temperature range, only moisture is released from the antimicrobial components. Above 180 °C, decomposition begins. However, their thermal stability is enough to prevent their chemical structure from breaking down during powder coating processing and curing, as the low-temperature polyester powder coating process occurs at 115 °C, and curing is performed at 160 °C. If the antimicrobial components degraded, they might lose their bacterial-reducing capabilities. In our tests, we achieved very high bacterial reduction results (even over 99% for PLY and HAL/PLY), confirming their stability during the powder coating production and curing process.

### 3.2. Powder Coatings Characterization

As an antibacterial additive, biopolymers such as polylysine (PLY) and quaternized chitosan (CH-Q) were used. To improve the dispersibility of the biopolymer in the powder coating formulation, its compatibility with the polymer matrix, and its washing resistance, immobilization on halloysite was employed. High reactive saturated polyester resin GP 95,518 was used to obtain low-temperature curing powder coating. To initiate the curing reaction, a typical β-hydroxyalkyl-amide curing agent was employed. Coatings were cured at 160 °C for 50 min. During this process, the hydroxyl groups of the curing agents reacted with the carboxyl groups of the polyester resin, forming ester bonds. During this process, water is also released and evaporates during heating. As a result, cross-linked coatings were obtained and tested for visual, mechanical, and antibacterial properties. The properties of the obtained coatings are presented in Table 4.

The sample containing halloysite alone has the lowest roughness, which indicates its good compatibility and dispersibility in polyester matrix. The roughness values for samples containing biopolymers immobilized on halloysite were lower than only PLY or CH-Q based and unmodified reference sample. The decrease in roughness parameters for these samples indicates an increase in their homogeneity with the polyester matrix, which is a result of the immobilization of biopolymers on halloysite.

The tested coatings were characterized by high gloss, except for the sample containing polylysine, which was matte. The high gloss indicates good compatibility and dispersion of the modifier in the polyester matrix. The low gloss of the coating containing polylysine is associated with its higher roughness and indicates poorer compatibility with polyester.

The thickness of the tested coatings ranges from 60.1 to 70.5 μm (Table 4). According to Qualicoat’s technical standards, the minimum average thickness recommended for the organic topcoat of the coating system to protect the steel substrate should be 60 μm [34]. The thickness values of the tested coatings meet the requirements.

Adding polylysine resulted in a decrease in relative hardness compared to the polyester-based reference sample, likely because of their more flexible chains due to the presence of aliphatic segments in its structure. The same is true for chitosan. However, the hardness of the CH-Q sample was higher than that of PLY due to its stiffer chain. For HAL and HAL/PLY-containing samples, hardness is also at the same level compared to the reference. Hardness HAL/CH-Q sample was slightly higher than the other coating, due to the stiffer structure of CH-Q than PLY.

Adhesion to the steel surface was high, thanks to polar ester functional groups in the polyester chain. These groups interact electrostatically with the steel substrate, boosting adhesion.

Samples containing polylysine were less resistant to cupping, possibly because of its linear structure affecting the tribological properties of the crosslinked polyester matrix. However, this value was higher than 5 mm, which is consistent with the technical requirements for powder coatings [34]. The cuppings of other coatings were at a very high level.

The effect of adding an antibacterial agent on the color of the coating is visible when polylysine is added; the color change determined by the ΔE index in SCE mode is 8.4 ± 0.28. In this case, color perception is influenced by surface development resulting from micro-roughness. The color change compared to the coating without additives mainly concerns the L* and b* components, moving towards yellow. In the case of coatings with other antibacterial additives, the color change ΔE relative to the reference coating is insignificant and amounts below 3, which is significantly lower than in the case of using, for example, active silver agents on carriers such as montmorillonite and vermiculite [35].

The data presented show that the samples containing polylysine have very high antibacterial activity against *E. coli* and *S. aureus*. The reduction in *E. coli* was higher than that of *S. aureus*, suggesting that PLY is more effective against Gram-negative bacteria, likely due to differences in cell wall structure. PLY immobilized on halloysite more effectively reduces *E. coli* and *S. aureus* bacteria. This may be due to the enhancement of this effect by halloysite, which itself has moderate bacterial reducing capacity. CH-Q showed a weaker bacterial reduction than polylysine, at 48.03% for *E. coli* and 81.56% for *S. aureus*. Immobilization of CH-Q on halloysite enhances this effect. The higher antibacterial effectiveness of polylysine may be due to its nearly double content of active ammonium groups (15 wt.% of nitrogen in PLY compared to 8 wt.% in CH-Q), as well as the more favorable spherical shape of PLY particles, which allows better dispersion in the coating matrix. Similar differences in biocidal properties are observed in the case of biopolymers deposited on halloysite.

Ultrasonication is a common method used to activate layered and nanotubular clay minerals by changing their morphology and surface properties [36]. Using relatively short sonication times can reduce the agglomeration of halloysite particles and enhance the dispersion of aluminosilicate in polymer matrices. On the other hand, excessively long sonication times can lead to re-agglomeration [37].

In our research, we used a sonication time of less than 20 min to improve the antibacterial properties of the HAL/CH-Q compound, which showed only moderate effectiveness against both Gram-positive and Gram-negative bacteria. As expected, coatings with ultrasonically activated HAL/CH-Q exhibited higher colony reduction levels for both *E. coli* and *S. aureus* bacteria compared to the non-modified ones.

Under the influence of ultrasonic waves, halloysite particles undergo abrasion, breaking the mineral grains and creating defects in their structure that constitute centers of catalytic activity [38]. This can enhance both the dispersion of halloysites in the coating and the availability of iron cations, which are bactericidal through oxidative action.

In the case of halloysite/polylysine compounds, high antibacterial activity (over 99.99% reduction in both bacterial strains) is observed even without additional activation of the additive.

**Figure 5:**
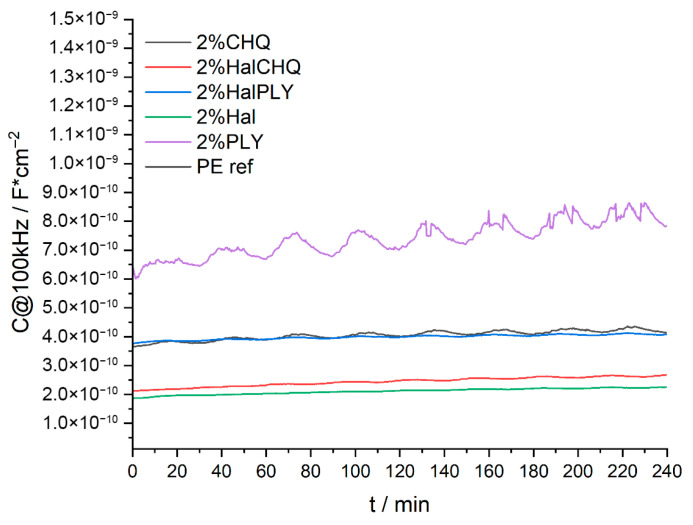
Capacitance (C@100 kHz) of polyester-based coatings measured during sinusoidal temperature cycling (20–50 °C).

**Figure 6:**
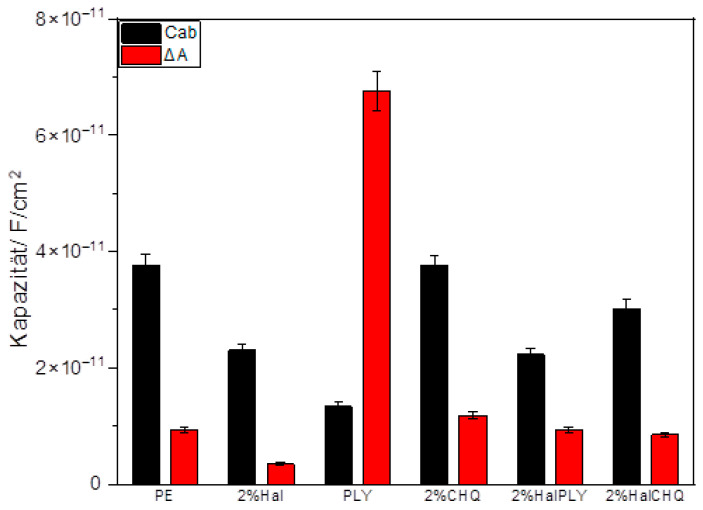
Average capacitance (C_ab_) and amplitude of cyclic capacitance variation (ΔA) for polyester-based coatings with different modifiers.

The capacitance of the coatings was monitored at 100 kHz during sinusoidal temperature cycling (20–50 °C) to evaluate reversible water uptake behavior. Figure 5 shows the changes in coating capacitance as a function of time, while Figure 6 compares the average capacitance values (C_ab_) and the amplitude of capacitance variation (ΔA) for all samples.

The polyester-based reference coating (PE ref) showed the lowest capacitance, indicating low water permeability and good barrier properties.

The coating containing polylysine (PLY) had a slightly higher capacitance, which may be related to the presence of hydrophilic functional groups in the biopolymer. Although this behavior suggests higher water sorption, it may also enhance the bioactive performance of PLY observed in antimicrobial tests.

Halloysite-containing coatings (HAL) displayed capacitance values like those of the reference, confirming good dispersion and compatibility with the polymer matrix. For the hybrid system with polylysine immobilized on halloysite (HAL/PLY), the capacitance decreased compared to the coating with PLY alone, indicating that immobilization on halloysite reduces water uptake and enhances coating compactness while preserving biofunctionality.

For quaternized chitosan (CH-Q), moderate capacitance values were observed. When CH-Q was immobilized on halloysite (HAL/CH-Q), a further decrease in capacitance was observed, suggesting that immobilization also enhances barrier performance. The overall amplitude of cyclic capacitance change (ΔA) followed the same trend, confirming reduced reversible water uptake for halloysite-based hybrid coatings.

**Figure 7:**
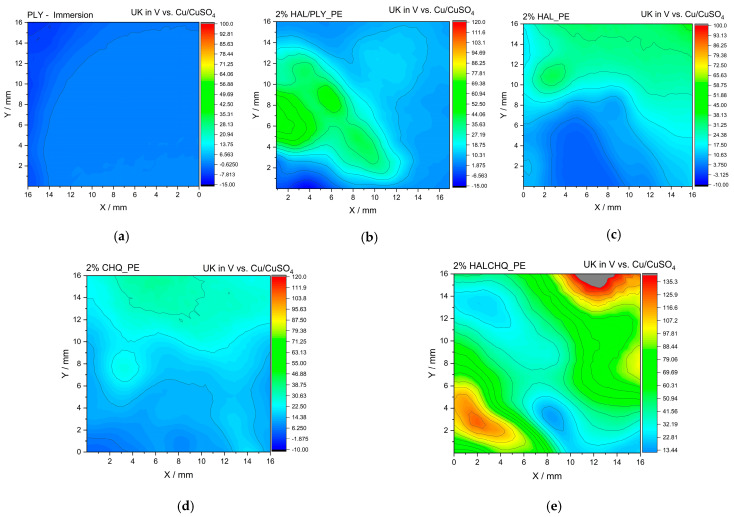
Surface potential maps of polyester-based coatings obtained by SKP (Cu/CuSO_4_ reference): (**a**) polylysine (PLY), (**b**) polylysine immobilized on halloysite (HAL/PLY), (**c**) halloysite (HAL), (**d**) quaternized chitosan (CH-Q) and (**e**) quaternized chitosan immobilized on halloysite (HAL/CH-Q). Identical potential scales were used for comparison of surface uniformity.

The surface potential distribution of the cured coatings was analyzed using a Scanning Kelvin Probe (SKP) technique to evaluate their electrochemical surface uniformity and local charge behavior. The measurements were performed under ambient conditions using Cu/CuSO_4_ as the reference electrode. Figure 7 presents the potential maps of the investigated coatings obtained at identical scale settings for direct comparison.

Along with the qualitative SKP maps, a quantitative summary of the surface potential values was included to support interpretation. The mean ± SD values were PLY: 0.002 ± 0.0012 V, CH-Q: 19.48 ± 7.49 V, HAL: 17.35 ± 1.69 V, and HAL/CH-Q: 61.91 ± 3.08 V. These results confirm the visual SKP maps and highlight the strong increase in positive surface potential for the halloysite–biopolymer hybrids, especially HAL/CH-Q.

The surface potential maps obtained by Scanning Kelvin Probe (SKP) measurements (Figure 7) revealed clear differences in surface electrochemical behavior among the tested coatings. The reference polyester coating (PE) and the coating containing only halloysite (HAL) exhibited homogeneous potential distribution with moderate potential values, indicating compact and uniform surfaces with good barrier properties.

In contrast, the coating containing polylysine (PLY) showed a more uniform but less electrochemically active surface, consistent with its insulating and bifunctional character. The incorporation of halloysite modified with polylysine (HAL/PLY) resulted in a distinct increase in potential variation across the surface, suggesting improved electrochemical activity and a more heterogeneous surface morphology, likely related to localized interactions between the immobilized biopolymer and the matrix.

For coatings containing quaternized chitosan (CH-Q) and its halloysite hybrid (HAL/CH-Q), pronounced potential gradients were observed, particularly in the HAL/CH-Q sample, which exhibited higher surface potential regions. This behavior indicates increased surface charge distribution caused by quaternary ammonium groups and the synergistic effect of halloysite, which enhances electrostatic interactions and improves surface reactivity and antibacterial function.

Overall, SKP analysis confirmed that immobilization of bioactive components on halloysite modifies the surface potential landscape, reflecting enhanced interfacial interactions and functional activity while maintaining the general compactness of the coating.

**Figure 8:**
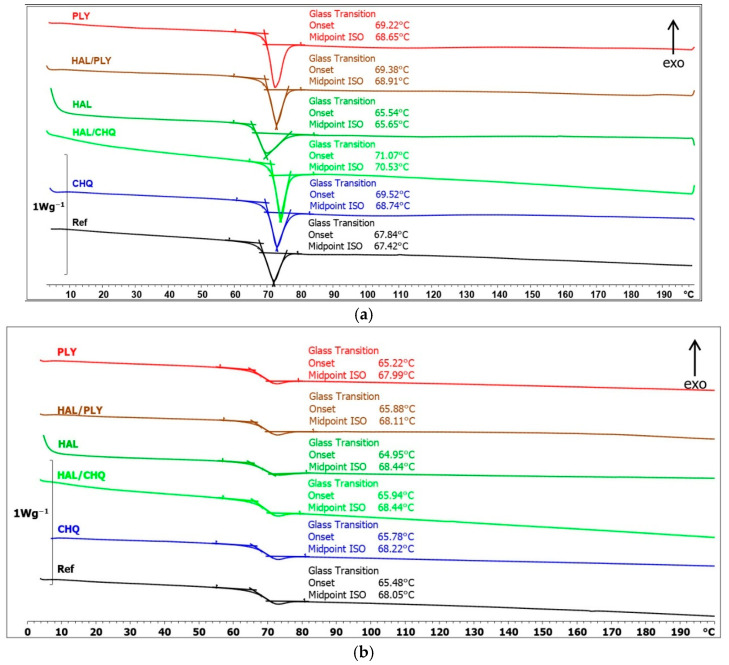
DSC curves recorded during (**a**) the first heating, (**b**) the second heating.

**Figure 9:**
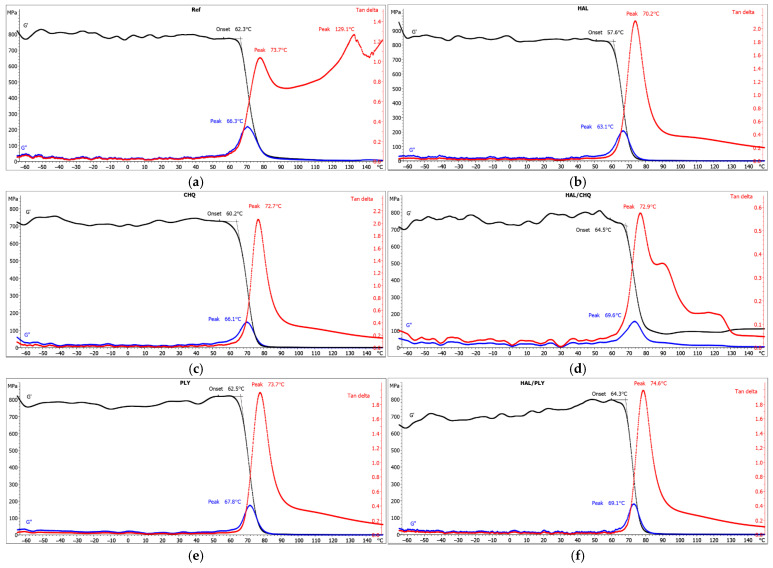
DMA curves of the samples: (**a**) reference without microbial additives, (**b**) with halloysite (HAL), (**c**) with quaternized chitosan (CH-Q), (**d**) with quaternized chitosan intercalated on halloysite (HAL/CH-Q, (**e**) with polylysine (PLY), (**f**) with polylysine intercalated on halloysite (HAL/PLY).

The effect of antimicrobial agents on the behavior of powder coatings during controlled heating was analyzed using DSC and DMA methods. (Figure 8 and Figure 9). In the DSC curves recorded during the first heating of the samples, a glass transition with relaxation was observed (Figure 8a). The glass transition temperature for the PE reference sample was 67.4 °C. For samples with 2 wt.% of additives, the inclusion of biocide compositions PLY, HAL/PLY, CH-Q, and HAL/CH-Q had little impact on the glass transition temperature, which ranged from 68.7 °C to 70.5 °C. Notably, the addition of 2 wt.% halloysite clearly lowered the glass transition temperature of the PE binder by 2 °C compared to the reference sample.

The glass transition temperature of the reference sample, determined from the DSC curve recorded at the second gasification stage, was 68.1 °C. For the other tested samples (Figure 8b), the determined glass transition temperature remained unchanged. The very slight differences in the values are due to the removal of thermal history and stresses in the tested coating samples. The DSC curves show the glass transition of the polyester resin without affecting the other components of the tested coatings. The thermal properties of CH-Q and HAL/CH-Q coatings are not impacted by quaternized chitosan.

Dynamic mechanical analysis revealed that the glass transition was the main transition observed during the tests. The peak value of the mechanical loss factor (tan δ) was used to determine the glass transition temperature. For the reference sample, the glass transition was found at 73.7 °C (Figure 9a). Very similar values were obtained for the CH-Q, HAL/CH-Q PLY, and HAL/PLY samples in the temperature range from 72.7 °C to 74.6 °C (Figure 9c–f). The lowest glass transition temperature was 70.2 °C for the sample containing 2 wt.% HAL (Figure 9b). This trend for the glass transition temperature is consistent with the DSC results from the first heating run. The HAL sample exhibited the highest intensity of the tan δ peak, indicating the least favorable damping properties of the tested coatings. The behavior of the HAL/CH-Q sample was different, with the lowest tan δ peak intensity, which suggests good damping properties. The reference sample displayed an intermediate peak intensity of tan δ. The other samples exhibited slightly lower tan δ intensities than HAL. Analysis of the storage modulus also reveals important features.

It can be concluded that the samples underwent relaxation and intramolecular reorganization of the PE matrix at an early stage of the test, followed by vitrification. At higher temperatures, the samples were in an elastic state, as evidenced by the low storage modulus value. The impact of the immobilized antimicrobial agents HAL/CH-Q and HAL/PLY is noticeable. The peak loss modulus value for these two samples was the highest among all tested. The storage modulus of the samples was similar, indicating their high energy storage capacity and are stiff during dynamic shear deformation. The HAL sample exhibited the lowest storage modulus onset value, which aligns with the measured glass transition temperature of this sample (as indicated by the tan delta peak), being the lowest among those tested.

**Figure 10:**
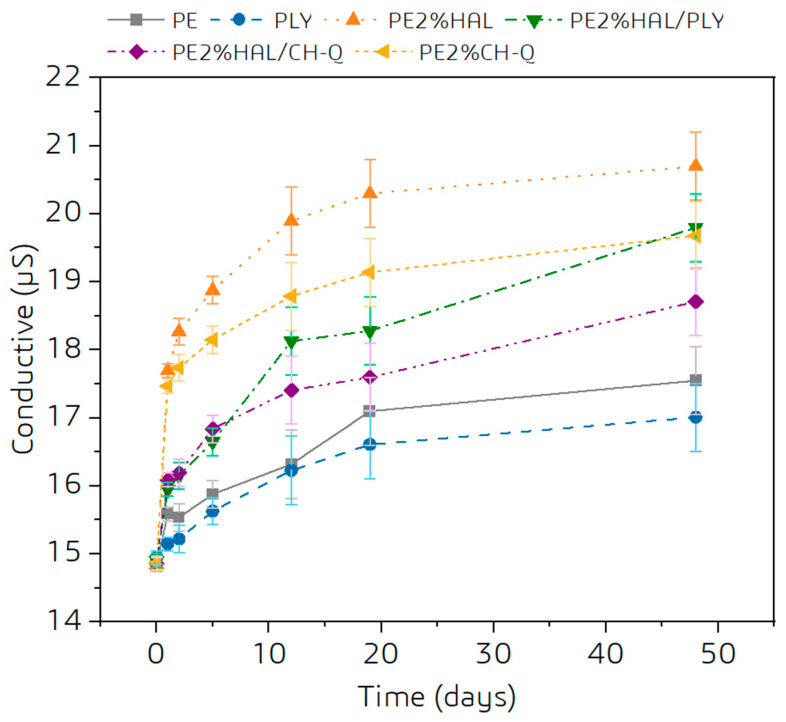
Conductometric analysis of washing resistance of polyester powder coatings with PLY, HAL, and CH-Q.

To initially evaluate the resistance of powder coatings to leaching of antibacterial additives, we monitored changes in water conductivity over time when the cured coating was in contact with water (Figure 10). The greatest increase in conductivity, about 15% over 2 weeks, was observed for the coating modified with halloysite alone. A rapid increase in conductivity was also observed for coatings containing quaternized chitosan. The concentration of dissolved electrolyte increases gradually for polylysine.

### 3.3. Structure–Performance Correlation (SEM, FTIR, SKP, WUR)

SEM analysis revealed that ε-polylysine (PLY) forms irregular spherical aggregates that show limited compatibility with the polyester matrix. This poor integration leads to reduced gloss, increased roughness, and a visible color shift compared to the reference coating. These effects were notably reduced when PLY or CH-Q were immobilized on halloysite. Although halloysite appears as larger blocks (Figure 3e) and agglomerates <5 µm (Figure 3b), it was well dispersed within the coating and did not adversely affect the mechanical or visual properties of the polyester system. Both PLY and CH-Q mainly interact with halloysite through electrostatic adsorption of positively charged amino or ammonium groups onto the negatively charged SiO_2_ surface, further stabilized by hydrogen bonding [39,40]. Quaternization introduces N^+^(CH_3_)_3_ groups into the chitosan chain, which enhances antibacterial activity [41] and increases the effective surface area of the particles (Figure 3d), but also decreases compatibility with the polymer matrix. FTIR confirmed the successful deposition of CH-Q on halloysite, consistent with the morphology observed in SEM.

Improved dispersion of HAL/PLY and HAL/CH-Q in the polyester matrix reduced local surface defects and resulted in a more homogeneous Volta potential distribution in SKP maps. Hybrid coatings exhibited fewer high-contrast regions, indicating decreased water adsorption sites and reduced susceptibility to interfacial micro-heterogeneities. Mechanistically, this corresponds to a denser, more uniform coating structure with improved resistance to ionic and moisture transport.

The coating containing PLY alone showed increased capacitance and higher ΔA values, confirming greater reversible water absorption. Immobilizing PLY on halloysite (HAL/PLY) decreased both capacitance and ΔA, indicating suppressed water uptake and improved barrier properties while maintaining biofunctionality. For CH-Q, moderate capacitance levels were observed, while HAL/CH-Q demonstrated a further decrease, showing that immobilization also enhances the barrier performance of quaternized chitosan. Lower capacitance was linked to reduced leaching and better antibacterial effectiveness. Overall, the combined SEM–FTIR–SKP–WUR analysis shows that immobilizing PLY and CH-Q on halloysite improves compatibility with the polyester matrix, reduces water absorption, increases structural density, and stabilizes antibacterial activity through better dispersion and fewer additives leaching.

## 4. Conclusions

Silver-free polyester powder coatings containing the bio-based antimicrobials polylysine (PLY) and quaternized chitosan (CH-Q) were successfully developed. Both biopolymers, either added directly or immobilized on halloysite nanotubes, provide effective antibacterial activity, with halloysite–biopolymer hybrids demonstrating the strongest overall performance. The incorporation of these additives did not weaken mechanical or thermal properties, and halloysite helped improve dispersion and surface uniformity.

These results demonstrate the potential of biodegradable, metal-free systems as sustainable options to silver-based antimicrobial coatings. Future research should emphasize long-term stability, leaching behavior, and optimizing halloysite–biopolymer interactions to further develop environmentally friendly antimicrobial powder coatings.

## 5. Patents

Pilch-Pitera, B.; Krawczyk, K.; Kędzierski, M.; Zubielewicz, M.; Kunce, I. et al., Antimicrobial powder paint based on polyester resin and method for producing antimicrobial powder paint based on polyester resin, Polish Patent Application P-453501 2025.

## Figures and Tables

**Table 1:** Qualitative/quantitative composition of powder coatings.

Component/ Symbol of Coating	Polyester Resin	XL-552	Benzoin	Byk 368P	Bayferrox 654	Albasoft 100	PLY	HAL	HAL/ PLY	CH-Q	HAL/ CH-Q
	[wt.%]										
PE (reference sample)	65.55	3.45	0.5	1.0	4.50	25.00	-	-	-	-	-
PLY	65.55	3.45	0.5	1.0	4.50	23.00	2.00	-	-	-	-
HAL	65.55	3.45	0.5	1.0	4.50	23.00	-	2.00	-	-	-
HAL/PLY	65.55	3.45	0.5	1.0	4.50	23.00	-	-	2.00	-	-
CH-Q	65.55	3.45	0.5	1.0	4.50	23.00	-	-	-	2.00	-
HAL/CH-Q	65.55	3.45	0.5	1.0	4.50	23.00	-	-	-	-	2.00

**Table 2:** Elemental analysis results of AA agents.

Symbol	C [wt.%]	H [wt.%]	N [wt.%]
PLY	39.87 ± 0.21	8.03 ± 0.04	15.00 ± 0.22
HAL	1.13 ± 0.07	1.81 ± 0.02	0.69 ± 0.01
HAL/PLY	13.38 ± 0.08	3.73 ± 0.01	5.22 ± 0.03
CH-Q	41.08 ± 0.03	8.23 ± 0.02	8.00 ± 0.03
HAL/CH-Q	15.81 ± 0.03	3.99 ± 0.02	3.34 ± 0.01

**Table 3:** TGA results of used components.

AA Symbol	Decomposition Temperature Ranges [°C]/wt.% Loss	DTG Max [°C]	Total wt.% Loss at 600 °C
PLY	25–180/8 240–560/83	76 310; 425; 444	91
HAL	25–200/2.5 200–700/12.5	80 480	15
HAL/PLY	25–180/3 180–600/36	80 312; 465	39
CH-Q	25–180/10 180–600/65	75 272; 408	75
HAL/CH-Q	25–180/4 180–600/29	72 274; 490	33

**Table 4:** Specification of polyester powder coatings properties.

Symbol of Coating		PE (Refer. Sample)	PLY	HAL	HAL/PLY	CH-Q	HAL/CH-Q
Roughness ISO 12085 [20]	Ra, µm	0.26 ± 0.02	**2.97 ± 0.02**	**0.09 ± 0.02**	**0.14 ± 0.03**	**0.48 ± 0.03**	0.24 ± 0.03
	Rz, µm	0.70 ± 0.16	**12.10 ± 0.12**	0.69 ± 0.17	0.83 ± 0.18	**2.13 ± 0.18**	0.77 ± 0.13
Gloss (60°) ISO 2813 [21]	GU	87.0 ± 1.2	**10.7 ± 1.5 **	**89.2 ± 0.9**	**84.5 ± 1.6**	**83.3 ± 1.1**	85.5 ± 1.2
Thickness ISO 2808 [22]	µm	60.1 ± 1.6	63.3 ± 2.5	65.4 ± 2.1	70.5 ± 1.8	61.7 ± 2.2	69.7 ± 2.6
Relative hardness ISO 1522 [24]	-	0.82 ± 0.02	**0.61 ± 0.02**	**0.78 ± 0.03**	0.82 ± 0.03	**0.69 ± 0.02**	**0.88 ± 0.03**
Adhesion to the steel ISO 2409 [23]	0-best 5-worst	0	0	0	0	0	0
Cupping ISO 1520 [25]	mm	10.01 ± 0.07	**6.37 ± 0.09**	**10.45 ± 0.11**	**9.08 ± 0.05**	10.06 ± 0.09	**10.47 ± 0.13**
Color ISO 7724 [26]	-	L* = 17.46 a* = 15.53 b* = 18.27	L* = 25.86 a* = 11.34 b* = 10.35	L* = 18.88 a* = 15.27 b* = 17.33	L* = 19.64 a* = 15.15 b* = 17.38	L* = 19.51 a* = 14.73 b* = 16.32	L* = 19.09 a* = 14.95 b* = 16.53
		-	ΔE*_ab_ = 8.40 ± 0.28	ΔE*_ab_ = 1.74 ± 0.58	ΔE*_ab_ = 2.38 ± 0.15	ΔE*_ab_ = 2.93 ± 0.07	ΔE*_ab_ = 2.44 ± 0.02
Reduction in *Escherichia coli* ISO 22196 [10]	%		99.9836 ± 0.0007	50.5226 ± 0.2223	99.9998 ± 0.0001	48.0256 ± 0.4550	50.2323 ± 0.3701; 70.9986 ± 0.1273(s)
Reduction in *Staphylococcus aureus* ISO 22196 [10]	%		99.9333 ± 0.0011	uncountable	99.9993 ± 0.0002	81.5603 ± 0.6282	70.9220 ± 0.0691; 98.6500 ± 0.0021(s)

In bold font: differences between the mean values based on *t*-test are statistically significant **(*p* < 0.05)**, highly significant **(*p* < 0.01)** (underline) or very highly significant **(*p* < 0.001)** (double underline), (s) HAL/CH-Q compounds are additionally sonicated.The surface roughness of the coatings was measured to characterize their topography. The sample containing polylysine showed the highest roughness parameters, which indicates a decrease in homogeneity in this coating, resulting from the difference in interactions between the resin and PLY particles related to their chemical structure. The sample containing quaternized chitosan alone also exhibited higher roughness parameters.

## Data Availability

The original contributions presented in this study are included in the article. Further inquiries can be directed to the corresponding authors.

## Abbreviations

The following abbreviations are used in this manuscript:

PLY	polylysine
CH-Q	quaternized chitosan
HAL	halloysite
MMT	montmorillonite
